# Supplementary material for: Occludin Acts as a Dynein Adaptor Regulating Permeability and Collateral Angiogenesis
Source: bioRxiv. 2025 Jun 15:2025.06.12.659326. Preprint. [Version 1] doi: 10.1101/2025.06.12.659326 (PMC12259183; doi:10.1101/2025.06.12.659326)
Supplement: 1 — Supplementary Figure 1. Expression of OCLN S490A and neovascularization in mice at 1 week after laser-induced choroidal neovascularization (CNV). (a) Representative epifluorescence images showing the laser-induced CNV lesions in the whole mount retinas stained with IB4 (purple). (b) Quantification of the lesion areas. Student t-test. P=0.2824. LM Ctrl: Tek-Cre−, Oclnfl3/fl3, OCLNS490A+/+; S490A: Tek-Cre+, Oclnfl3/fl3, OCLNS490A+/+. Supplementary Figure 2. Expression of OCLN S471 mutants reduced occludin binding to dynein LIC2. (a) U2OS cells were co-transfected with LIC2-FLAG, or pCMV-FLAG with GFP-WT or S471 OCLN mutants (S471D or S471A) for 48 hours. 3xFLAG tagged LIC2 was captured with anti-FLAG M2 mAb and protein G Sepharose beads then eluted by 3xFLAG peptide. The input lysates and IP elutes were IB with goat anti-turboGFP, mouse anti-FLAG M2 Abs. (b) Results of the quantification from IP experiments are expressed as the mean relative to the WT OCLN ± S.E.M. (c) Quantification of the percentage of transfected U2OS cells with γ-tubulin and OCLN colocalization. Black bars, complete colocalization; open bars, no colocalization. Results are expressed as the percentage in each group with Chi-Square analysis. ***P<0.001 and ****P<0.0001. Supplementary Figure 3. OCLN coiled-coil domain alone was not shown to interact with LIC2. U2OS cells were co-transfected with LIC2-HA and N-terminal GFP tagged wild type OCLN (GFP-WT, arrow) or GFP-coiled-coil region (GFP-CC, arrowhead) and IP with rat anti-HA mAb. The input lysates and IP elutes were IB with goat anti-GFP pAb, rat anti-HA mAb. [file NIHPP2025.06.12.659326V1-supplement-1.pdf]

Supplementary Figure 1

bioRxiv preprint doi: https://doi.org/10.1101/2025.06.12.659326; this version posted June 15, 2025. The copyright holder for this preprint (which was not certified by peer review) is the author/funder. This article is a US Government work. It is not subject to copyright under 17 USC 105 and is also made available for use under a CC0 license.

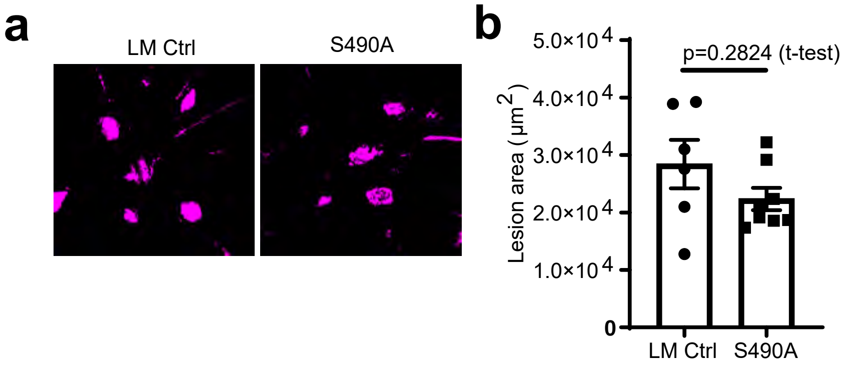

Supplementary Figure 2

bioRxiv preprint doi: https://doi.org/10.1101/2025.06.12.659326; this version posted June 15, 2025. The copyright holder for this preprint (which was not certified by peer review) is the author/funder. This article is a US Government work. It is not subject to copyright under 17 USC 105 and is also made available for use under a CC0 license.

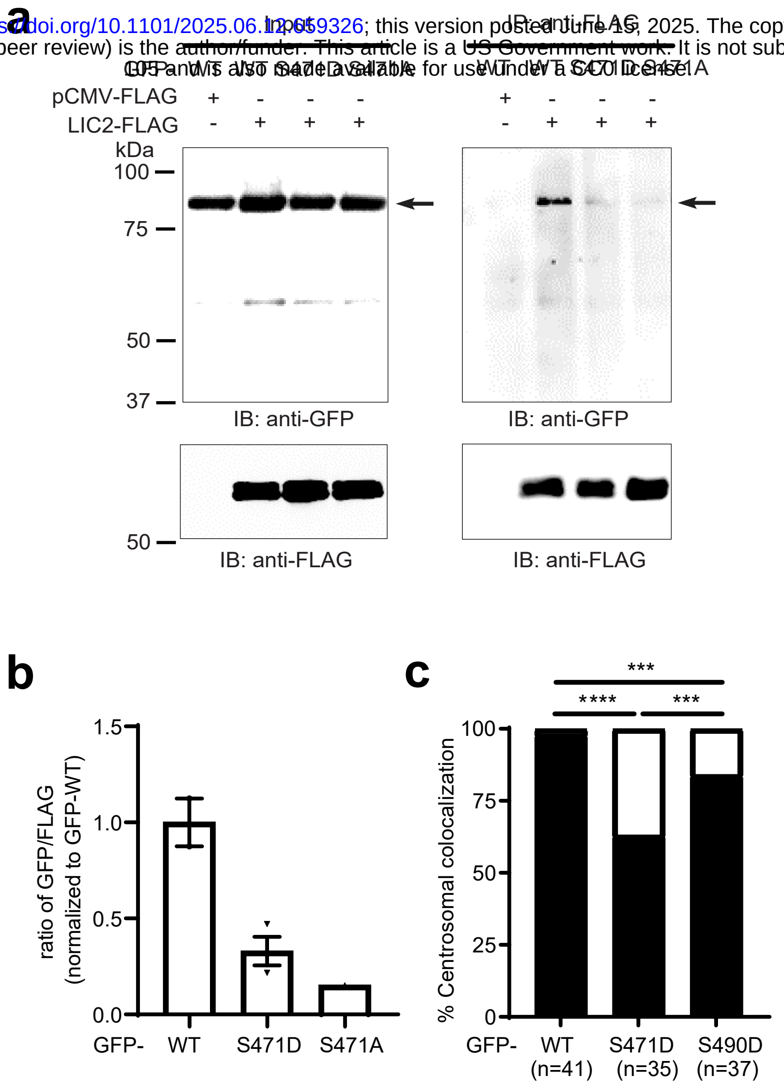

Supplementary Figure 3

bioRxiv preprint doi: https://doi.org/10.1101/2025.06.12.659326; this version posted June 15, 2025. The copyright holder for this preprint (which was not certified by peer review) is the author/funder. This article is a US Government work. It is not subject to copyright under 17 USC 105 and is also made available for use under a CC0 license.

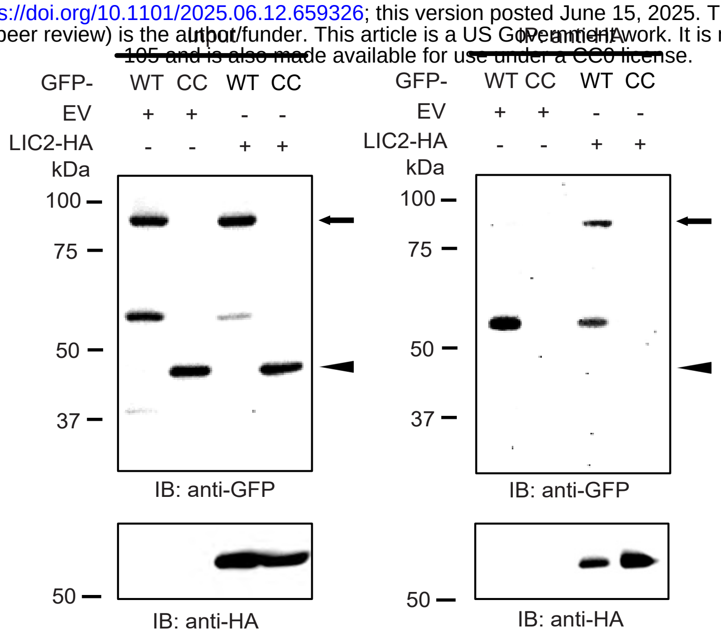

bioRxiv preprint doi: https://doi.org/10.1101/2025.06.12.659326; this version posted June 15, 2025. The copyright holder for this preprint (which was not certified by peer review) is the author/funder. This article is a US Government work. It is not subject to copyright under 17 USC 105 and is also made available for use under a CC0 license.

Supplementary table

Reagent type	Designation	Source or reference	Identifiers	Additional information
Cell line (*Homo-sapiens*)	human osteosarcoma U2OS cell line	ATCC	Cat#: HTB96	
Antibody	Anti-Mouse CD31 (Rat monoclonal)	BD Biosciences	Cat#: ab553370	IF (1:100)
Antibody	Anti-CD31/PECAM-1 (Goat polyclonal)	R & D Systems	Cat#: AF3628	IF (1:100)
Antibody	Anti-Ki67 (Rb polyclonal)	Abcam	Cat#: ab15580	IF (1:100)
Antibody	Anti-Occludin (Ms monoclonal)	Thermo Fisher	Cat#: 33-1500	IF (1:200); WB (1:1000)
Antibody	Anti-Occludin (Rb polyclonal)	Thermo Fisher	Cat#: 71-1500	IF (1:200); WB (1:1000)
	Anti-Occludin (Rb polyclonal)	Abcam	Cat#: ab167161	IF (1:200); WB (1:1000)
Antibody	Anti-Gamma-Tubulin (Ms monoclonal)	Sigma	Cat#: T6557	IF (1:200); WB (1:1000)
Antibody	Anti-Tubulin antibody [YL1/2]	Abcam	Cat#: ab6160	IF (1:400)
Antibody	Anti-V5 (Rb polyclonal)	Sigma	Cat#: AB3792	IF (1:100); WB (1:1000)
Antibody	Anti-Dynein (Ms monoclonal)	Sigma	Cat#: MAB1618	IF (1:100); WB (1:1000)
Antibody	Anti-DYNC1LI2/LIC2	Abcam	Cat#: ab178702	WB (1:1000)
Antibody	anti-Dynectin subunit 1/p150 [Glued] (Ms monoclonal)	BD Biosciences	Cat#: 610474	IF (1:200); WB (1:1000)
Antibody	Anti-NuMA (Ms monoclonal)	Sigma	Cat#: SAB2702236	WB (1:1000)
Antibody	Anti-CEP164 (Ms monoclonal)	Santa Cruz	Cat#: sc-515403	IF (1:100)
Antibody	Anti-percentrin (Rb polyclonal)	Abcam	Cat#: ab4448	IF (1:100)
Antibody	Anti-TurboGFP(d)	Thermo Fisher	Cat#: NC0461765	IF (1:100); WB (1:1000)
Antibody	Anti-HA High Affinity (Rat Monoclonal)	Roche	Cat#: 1867423	WB (1:10000)
Antibody	Anti-FLAG M2 (Ms monoclonal)	Sigma	Cat#: F1804	WB (1:10000)
Antibody	Rabbit anti-Occ pS490 pAb			IF (1:250)
Plasmid	RFP-pericentrin	Gift of Dr.Sean Munro (University of Cambridge, Cambridge, United Kingdom)		
Commercial assay or kit	Click-i EdU Alexa Fluor 594 Imaging Kit	Thermo Fisher	Cat#: C10339	
Commercial assay or kit	Click-iT Plus TUNEL Assay, Alexa Fluor 647	Thermo Fisher	Cat#: C10619	
Commercial assay or kit	HCAEC Nucleofector Kit	Lonza	Cat#: VVPB-1001	
Commercial assay or kit	Pierce BCA assay	Thermo Fisher	Cat#: 23225	
Other	Isolectin IB4 Alexa Fluor 488 conjugate	Thermo Scientific	Cat#: I21411	
Other	Isolectin IB4 Alexa Fluor 647 conjugate	Thermo Scientific	Cat#: I32450	
Other	Corning Collagen I, Rat	Thermo Scientific	Cat#: CB-40236	
Other	MEM EAGLE D VAL MOD W LGLT	Thermo Scientific	Cat#: NC9930008	
Other	Gibco™ MEM Vitamin Solution (100X)	Thermo Scientific	Cat#: NC9930008	
Other	Corning™ Endothelial Cell Growth Supplement (ECGS)	Thermo Scientific	Cat#: CB-40006B	
Other	Collagenase type I	Thermo Scientific	Cat#: NC9482366	
Other	Deoxyribonuclease I	Thermo Scientific	Cat#: NC9131722	
Other	Dispase	Thermo Scientific	Cat#: NC9199795	
Other	VeriBlot for IP Detection Reagent (HRP)	Abcam	Cat#: ab131366	
Other	Tylosin solution	Sigma	Cat#: T3397	
Other	MCDB 131 Medium	Sigma	Cat#: M8537	
Other	F1141 Fibronectin from bovine plasma - solution	Sigma	Cat#: F1141	
Other	Water, sterile-filtered, BioReagent, suitable for cell culture	Sigma	Cat#: W3500	
Other	Heparin Sodium (Laboratory), Fisher Chemical,Amber Glass; 100,000 units	Thermo Scientific	Cat#:H19	

bioRxiv preprint doi: https://doi.org/10.1101/2025.06.12.659326; this version posted June 15, 2025. The copyright holder for this preprint (which was not certified by peer review) is the author/funder. This article is a US Government work. It is not subject to copyright under 17 USC 105 and is also made available for use under a CC0 license.

Other	Heparin sodium salt from porcine intestinal mucosa	Sigma	Cat#:H3149	
Other	ENDOGRO (100mg)	VEC TECHNOLOGIES, INC.		
Other	McCoy's 5A Medium	ATCC	Cat#: 30-2007	
Other	Antibiotic Antimycotic Solution (100×)	Thermo Scientific	Cat#: 15240062	
Other	8-well chmabered coverglass	Thermo Scientific	Cat#: 155409	
Other	Hoechst-33142 (10 mg/ml)	Thermo Scientific	Cat#: H3570	
Other	Lonza Walkersville EBM MEDIUM SERUM FREE	Thermo Scientific	Cat#: NC9230063	
Other	35 mm Dish, No. 1.5 Coverslip, 20 mm Glass Diameter	MatTek Corporation	Cat#: P35G-1.5-20-C	
Other	µ-Dish 35 mm, high	iBIdi	Cat#: 81156	
Other	18 mm Coverslip, No. 1.5 Glass Thickness	MatTek Corporation	Cat#: PCS-1.5-18	
Other	ProLong™ Glass Antifade Mountant, 10 ml	Thermo Fisher	Cat#: P36984	
Peptide, recombinant protein	R&D SYSTEMS INCRHVEGF 165 (50ug)	Thermo Scientific	Cat#: 293VE050	
Peptide, recombinant protein	Epidermal Growth Factor human	Sigma	Cat#: E9644	
